# The expectations of generation Z regarding the university educational act in Romania: optimizing the didactic process by providing feedback

**DOI:** 10.3389/fpsyg.2023.1160046

**Published:** 2023-09-29

**Authors:** Mihaela Laura Bratu, Lucian-Ionel Cioca, Raluca Andreea Nerisanu, Mihaela Rotaru, Roxana Plesa

**Affiliations:** ^1^Department of Industrial Engineering and Management, Faculty of Engineering, Lucian Blaga University of Sibiu, Sibiu, Romania; ^2^Department of Technical Sciences, Academy of Romanian Scientists, Bucharest, Romania; ^3^Department of Accounting and Finance, Faculty of Economic Sciences, Lucian Blaga University of Sibiu, Sibiu, Romania; ^4^Faculty of Science, Department of Social Sciences, University of Petroşani, Petrosani, Romania

**Keywords:** feedback, generation Z, educational management, engineering, SKS method, educational innovation

## Abstract

In the university education system, the evaluation and provision of two-way teacher–student feedback are tools within the control function of educational management. Feedback can be defined as the process that takes place in the context of teacher–student interaction during university courses, both individually and in groups, for the development and achievement of the performance of the two actors involved through evaluation, appreciation, support, perception, and teaching. The research aims to develop an innovative feedback tool for the higher education engineering sector to support the improvement of learning outcome-oriented curricula and teaching activities to better meet the learning needs of Gen Z students while being relevant to the labor market and to society in general. The research had a number of subjects: 246 students (67.5% women, 32.5% men) and 7 teachers to whom two feedback instruments were applied (the SKS instrument and the standard instrument of Lucian Blaga University of Sibiu). After testing the four hypotheses, it was observed that the feedback provided by Gen Z is focused on four areas of competence: psychological, pedagogical, education management, and general impression. Each field includes a set of professional and transversal competencies. The SKS (STOP, KEEP, and START) evaluation form is more reliable in evaluating different disciplines than the standard evaluation form by providing a more homogenous type of feedback for each discipline or teacher.

## 1. Introduction

Educational management is the science and art of preparing human resources, forming personalities according to some goals requested by society and accepted by the individual, which is necessary to be efficient and productive in educational relations, and stimulating transformation at the level of personalities, of both students and teaching staff (Tudorica, 2006). The evaluation and provision of two-way teacher-student feedback are tools within the control function of educational management (Tuturea et al., 1997; Crețu and Nicu, 2004). Accountability in the educational process at the university level establishes a strong foundation for today’s students, enabling future engineers to solve tomorrow’s problems.

By examining the feedback process from students to teaching staff, this article aims to optimize the operational management control function at the university level. The purpose of the research is to develop a feedback tool for teachers that directly reflects the current needs and interests of students and is used to improve the didactic act at the university level.

This article is structured as follows. In the following section, a literature review is conducted regarding the control function of educational administration, the evaluation and feedback provided by university students, and the essential characteristics of Generation Z. Section 3 presents methodological considerations regarding the paper’s methods, resources, and data. Section 4 results section is followed by the section 5 discussions, which offer similar studies in relation to the present findings, prospective directions, and current limitations. Conclusions and references are included in the final section.

## 2. Literature review

### 2.1. Educational management

A definition of educational management is provided by Iosifescu (2000), who proposes the definition of management as a starting point. In this context, educational management designates the science, art, and technique of planning, leading, organizing, and controlling the elements of a system in a specific field of activity. In our case, the organization is the university.

Three levels of educational management are distinguished. Macrostructural educational management, which is the third level, is referred to as strategic management, and it is showcased through national, European, and global educational policies at the education system’s level. This level has high generality. Level 2, the intermediate level, is carried out at the university level by the management team in the form of tactical management (Crețu and Nicu, 2004). Level 1 is represented by the educational management carried out by each teacher in the didactic activities of the student groups (operational management). The three levels differ in name, effective authority, formal elements, and the personality traits of the directly responsible persons, students and teaching staff, among which we mention knowledge, competencies, skills, attitudes, and values (Tudorica, 2006).

Educational management includes the design of the institutional network, the formulation of purposes and contents, training and professional development, the establishment of evaluation techniques that regulate the education system and processes along the way, and the optimization of results. Educational management, characterized by its systemic nature, transforms “inputs” into “outputs.” Through its indicative-instrumental function, it guides how to achieve objectives, adhere to principles, and apply methodologies (Tudorica, 2006). Educational management fulfills five functions that define, regulate, and optimize the educational process at the level of the organization: forecasting, organizing, coordinating, motivating, and controlling (Tuturea et al., 1997).

The control function in educational management involves the permanent and complete verification of how activities are carried out compared to the established standards and programs. Following verification, deviations from these standards and programs are identified, followed by pinpointing causes and suggesting corrective measures. The actions carried out are verification, tracking, regulation, monitoring, and evaluation of the process to find intelligent solutions and to improve some negative effects for the continuous improvement of the quality of the education process (Orțan, 2003). The evaluation and the provision of two-way teacher-student and student-teacher feedback are tools within the control function of educational management.

The evaluation of students’ knowledge and skills by teachers ensures the fulfillment of some essential educational goals in the university environment, establishing whether the objectives of the education system or process are achieved (Crețu and Nicu, 2004). The evaluation is an essential component of the educational process, providing direct information regarding the achievement of performance standards by students and, at the same time, providing indirect information regarding the teachers, the quality of the didactic act, the faculty, the university, and the educational system as a whole.

Evaluation is a complex action that includes measurement, judgment, and decision-making operations. Measurement involves assigning a number to an object or an event according to an accepted logical rule. Valuation involves making a value judgment on the result of a measurement. It is a qualitative evaluation that includes praise and critical remarks. The quality of an evaluation depends largely on the experience and personality traits of the evaluator (Crețu and Nicu, 2004), as well as on the degree of their docimological and pedagogical training (Mag, 2014; Nicu, 2015, 2017).

According to the National Education Law (Legea Nr. 1/2011, 2011) and the Magna Carta of the “Lucian Blaga” University of Sibiu (ULBS) (Universitatea “Lucian Blaga” din Sibiu, 2004), students have the right to participate, through the free expression of opinions, after a procedure approved by the university senate, in the evaluation of the activity for the subjects who attended. At the level of school legislation (Universitatea “Lucian Blaga” din Sibiu, 2004; Legea Nr. 1/2011, 2011), it was observed that the term feedback is not used, which is mainly used in the scientific community.

Feedback represents a retroaction that manifests itself at the level of different systems (biological, technical, etc.) to maintain their stability and balance against external influences: reverse feedback, reverse connection, circular causality, and closed causal chain (Academia Română, 2005). In the “Pedagogical Lexicon,” feedback is an action to inform the educator, through control and evaluation, of the results of his educational action (Nastas, 2019) to further take measures to improve the activity (Ştefan, 2006). Another pedagogy dictionary offers a three-dimensional perspective on the concepts of cybernetics, programmed education, and communication reports (Pieron, 2000; Schaub et al., 2001). In Andrei Cosmovici’s view, feedback is a “form of reverse connection, the way in which finality becomes causality,” but specifying that it is a “form of feedback also present in didactic communication” (Cosmovici, 2005), meaning that, in the literature of the sciences of education, it strengthens its position (Odolbeja, 1978; Judea, 2002; Cucoș, 2009).

It can be observed from the definitions above that assessment and feedback at the level of education have not only common points but also differences. Regarding purpose, feedback is a reaction to maintain balance, while evaluation is about measuring and judging. The form the feedback takes is descriptive, while the evaluation process is predominantly evaluative.

At the operational level, the evaluation process emphasizes measurement, followed by assessment and remediation. Feedback focuses more on assessment and remediation than measurement. Evaluation tends to focus on knowledge, skills, and abilities, while feedback can be given on a wider spectrum, including four categories: feedback about the task, feedback about the processing of the task, feedback about learner self-regulation, and feedback about self (Hattie and Timperley, 2016). At the same time, feedback can be positive or negative (Hattie and Timperley, 2016). For feedback to be effective, it is very important to give it in a place of trust and to be aware of its intention from the very beginning. Otherwise, it will be viewed as a criticism and nothing more.

In the scientific literature, there is a procedural approach to the two concepts, evaluation and feedback, from the perspective of optimization. The school represents a living organism, transformable from the perspective of optimization. From a macro perspective on education, optimization represents the successive passage of the domains: planning, implementation, evaluation, and feedback, according to the quality cycle, inspired by Deming’s circle (Nastas, 2019).

Process optimization is triggered when a result is obtained in the evaluation area, either due to a level below expectations or the desire for continuous improvement (Nastas, 2019). Therefore, feedback is a strategy that causes the best changes in a situation within the limits of available resources (Vaughn, 2003). It has been noted that evaluation and feedback are different processes that complement each other within the system and complete the loop.

### 2.2. Educational feedback

To provide a precise analysis of the feedback concept, following the latest developments in bibliographic research, a systematic literature review supported by bibliometric analysis based on the similarity visualization technique (Odett, 2022) was used.

To analyze the “feedback” topic, the bibliometric method of scientific mapping was used with the help of the visualization software VOS Viewer. The VOSviewer is widely used and has powerful graphical and mapping visualization capabilities. Bibliometric analysis has facilitated the mapping of large volumes of scientific literature, a method that guarantees the quality of the information used and the results generated (Al Husaeni and Nandiyanto, 2021).

The first step involved a comprehensive search of the Thomson Reuters Web of Science Core Collection (WOS) database, which is recognized as the most reliable database for bibliometric studies as it searches across publishers and shows no publisher bias (Ding et al., 2014). In addition, it guarantees the inclusion of the most important journals. Indeed, to ensure that the WOS results are of high quality, they were quantitatively limited. The database was considered the most suitable, following previous searches in feedback literature.

The sample included in the feedback analysis is based on articles indexed on the Web of Science database in English and includes 523,092 entries, of which 3,936 were selected based on language filters (English); Citation Topics Meso: education and educational research; Web of Science categories: education and educational research, and publication year: 2018–2022. Bibliometric analysis is useful for the purpose of the principle of classification of the research and the identification of thematic similarities.

The Web of Science sample was first exported in the required format and saved locally as a master file. Network analysis was performed with VOSviewer. A network comprises two formative elements: articles and links. Items represent the objects to be analyzed, e.g., publications, authors, or keywords. The connection between two items in the network examined in the context of the respective analysis is represented by links. This connection may refer to the co-occurrence of subject headings, a bibliographic link, co-authorship, etc. (van Eck and Waltman, 2018). Several functions ensure the highlighting of important structures in the data. The most important properties of items are their weights and cluster membership. Items with high weights are classified as significant and are therefore highlighted in the view with a larger circle. Group membership is expressed by colors and indicates a group of closely related elements (Odett, 2022). Spacing between items is also important. They are a rough graphical illustration of their connection strength.

This scientific literature mapping study used bibliometric methods to review research on feedback in education. Research evaluations based on bibliometric methods do not examine the substantive conclusions of the studies. Rather, their value extends from the ability to document and synthesize broad trends that describe the landscape, composition, and intellectual structure of a knowledge base (Odett, 2022).

**Figure 1:**
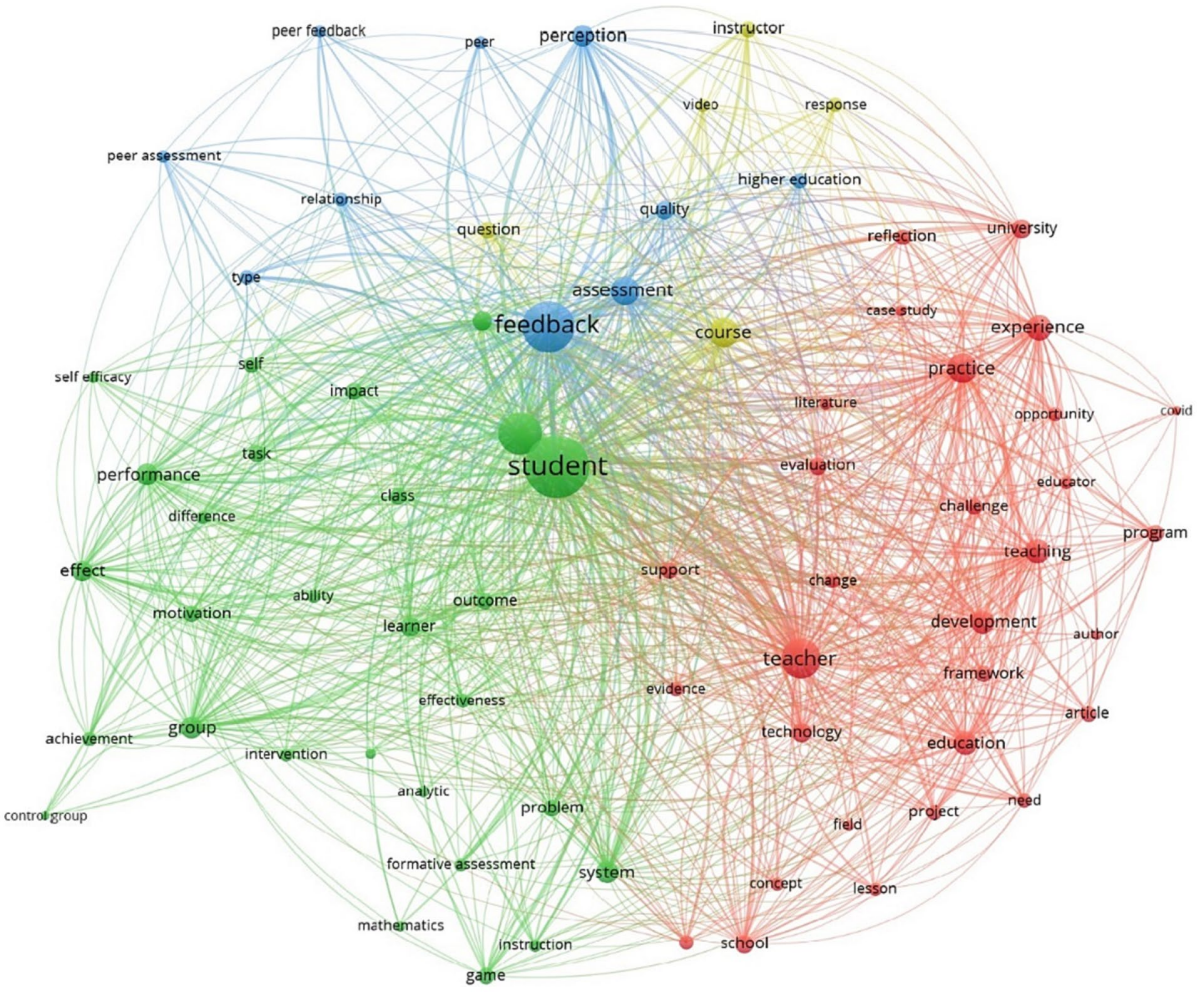
Keyword analysis on feedback in education (VOSviewer).

The keyword analysis of feedback in education essentially showed four clearly distinct main areas, as shown in Figure 1. The red cluster has the teacher as the central element. The green cluster is about the student-centered part of feedback. The blue cluster is centered on the notion of feedback, and the yellow cluster is central to the notion of course.

Cluster 1, teacher, has 30 keywords, among which we mention application, reflection, teacher, technology, university, challenge, change, education, development, evaluation, experience, need, opportunity, and practice. Cluster 2, student, includes 28 items, among which we mention ability, achievement, analytic, class, control group, difference, effect, engagement, formative assessment, game, group, learning process, motivation, outcome, performance, self-efficiency, and system. Cluster 3, feedback, includes 10 items: assessment, higher education, peer, perception, quality, relationship, and type. Cluster 4, course, has 5 items: course, instructor, question, response, and video.

Following the analysis of the clusters and keywords, it can be observed that the feedback at the level of education and research in the last 5 years has, at its center, the notion of the student, associated with the feedback and the notion of the professor. The keywords show the two actors of education: the student and the teacher, the process (the feedback), and the learning context (the course).

**Figure 2:**
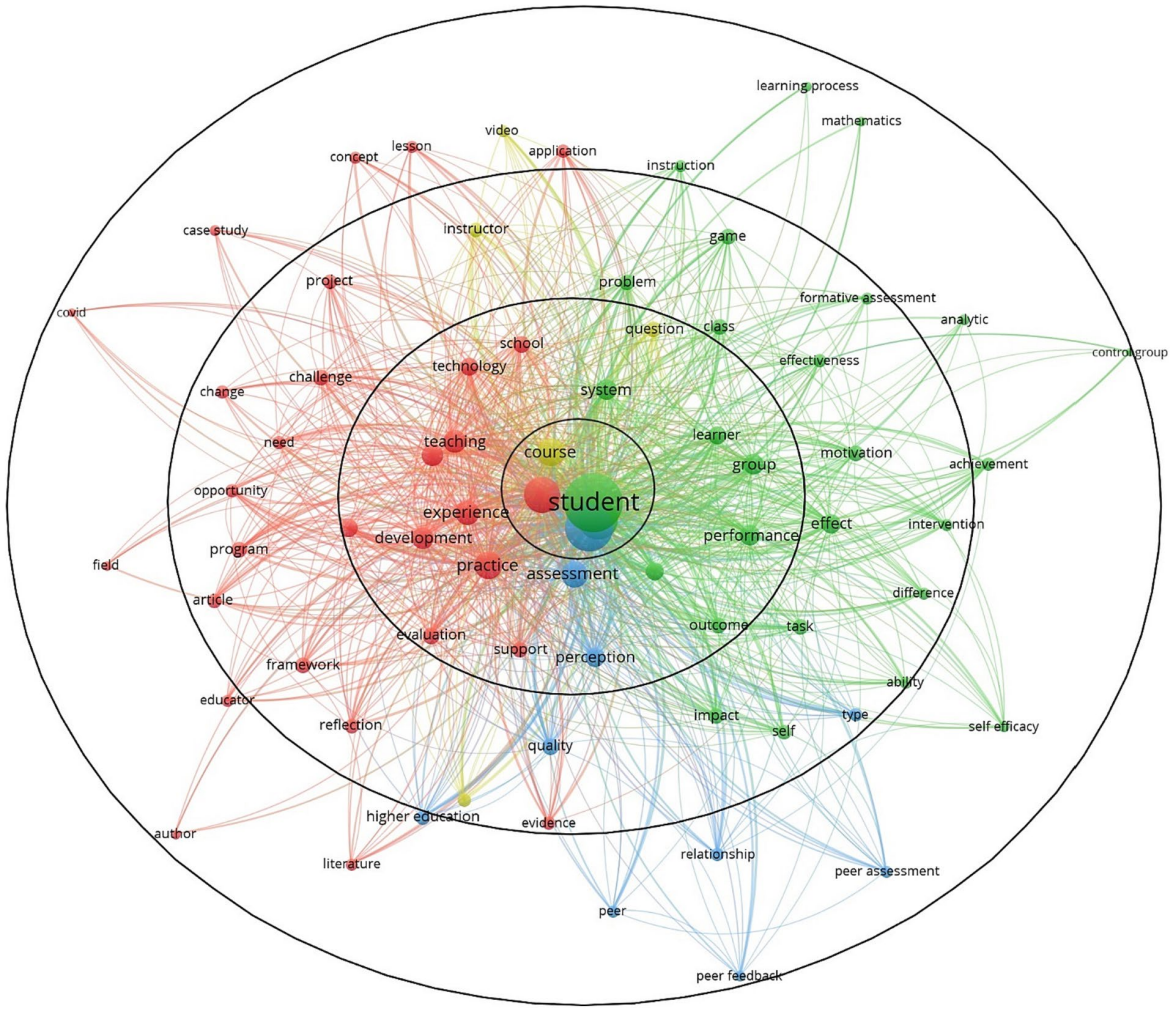
Feedback analysis through the fractionalization analysis method (VOSviewer).

When we change the analytical approach from normalization to fractionalization, we can see in Figure 2 that the four terms connected with the feedback process in education are student, feedback, instructor, and course. At level 2, feedback is defined by the following questions:

- Where? – the general context of the event, the university and the school, and the system.
- Who? – is carried out in individual and group contexts.
- How? – questions and technology.
- What is the purpose? – performance, experience, development, and practices.
- What is the method? – evaluation, assessment, support, perception, and teaching.

Level 2 of feedback is delimited by the second circle of Figure 2 and includes a series of terms that are answers to the above questions, deepening the understanding of the analyzed concept and widening the scope of the action of the feedback. The five questions define the concept from the spatial perspective of human, material, and procedural resources.

Level 3 of feedback (the third circle of Figure 2) captures the factors related to the notion of feedback for students: motivation, self, impact, effect, game, problem, and activity; for teachers, reflection, opportunity, challenge, change, and need; for feedback, quality and type; and for the course, it is the instructor.

In Figure 2, Level 3 of feedback (represented as the third circle) encompasses crucial factors pertaining to the concept of feedback. These factors are categorized as follows:

- For students: motivation, self-assessment, impact, effect, game, problem, and activity.
- For teachers: reflection, opportunity, challenge, change, and needs.
- Regarding feedback: quality and type.
- For the course: instructor-related aspects.

This framework organizes and delineates the components of the feedback process, allowing for a comprehensive analysis of its various elements and their interplay.

In conclusion, following the analysis of the concept of feedback through the bibliometric method of scientific literature mapping with the help of the VOSviewer visualization software, feedback can be defined as the process that takes place in the context of teacher-student interaction during school courses/ university, at individual and group levels, for the development and achievement of the performance of the two actors involved through evaluation, appreciation, support, perception, and teaching. For students, feedback is a method that affects motivation and self-image, producing effects in the context of activities, problem-solving, and games. For teachers, feedback is a need, an opportunity for reflection and introspection, and an opportunity and challenge for change.

While feedback is central to learning, research has largely been neglected, particularly from the student’s point of view (Mag, 2019). This gap in practice is particularly evident at the “Lucian Blaga” University of Sibiu, Faculty of Engineering. The university’s quality assurance department has developed a teacher evaluation tool for students, but does it not produce the desired effects? There are several problems associated with using the tool:

- Lack of student involvement in completing the questionnaire.
- The feedback form is only perceived as a formality by students and teachers.
- Questionnaire items do not provide sufficiently detailed feedback so that the teaching staff can improve their work.
- It does not produce effects at the level of the didactic act.
- The extent to which the reported aspects have been improved is not evaluated.

From an administrative point of view, the request and provision of feedback fall under the responsibility of the Quality Assurance Service. The mission of the Quality Assurance Service is to create a quality management system based on a policy, an organizational structure, and procedures that allow the control, evaluation/auditing, and continuous improvement of the quality of the entire university’s activities. Feedback as a reverse connection ensures the closing of the loop in education by ensuring the quality and increasing the sustainability of the educational process. Since 1992, sustainability has been included on the list of priorities of the United Nations of being a desire that refers only to the environment (Hayati et al., 2010; Menichini and Rosati, 2013; White and Noble, 2013; Metz et al., 2016; Natalia and Viorica, 2016; Rajiani and Kot, 2018; Bratu, 2019a,b; Smirnova et al., 2021; Bratu et al., 2022; Stanciu et al., 2022), but, currently, it covers all fields of activity, including education at the university level (Moss Gamblin, 2014; Stuart, 2015; Bratu and Cioca, 2018, 2019; Coleman and Gould, 2019; Emblen-Perry, 2019; Cristescu and Nerișanu, 2021; Cioca and Bratu, 2021a; Odett, 2022). Sustainability is the context in which university education takes place. The current problems of increased energy costs and the lack of integration of higher education graduates into the workforce in their field of study represent pressing issues regarding the use of resources. Along with these, feedback is a method that leads to the sustainability of education.

### 2.3. Generation Z

At the university level, there is a concern about adapting feedback tools to the ever-changing student generations. Currently, Generation Z comprises students and young adults.

Generation Z is the demographic cohort that succeeds Millennials (or Generation Y) and precedes Generation Alpha. The birth of those in this generation began in 1997 and ended in 2012. Most members of Generation Z have used digital technology from an early age and are familiar with the internet and social media, but they are not necessarily digitally literate (Iorgulescu, 2016; Francis and Hoefel, 2018; Prakash Yadav and Rai, 2020). Generation Z tends to be frugal and risk-averse compared to millennials, who tend to be more flexible at work. Despite having fewer instances of teenage pregnancies, alcohol consumption, and drug addiction than previous generations, Gen Z is frequently depicted in the media as anxious and depressed. Some researchers believe that these effects are due to the time spent on social networks and smartphones, the COVID-19 pandemic, and the recession caused by it (Turner, 2015; Dangmei and Singh, 2016; Shatto and Erwin, 2016; Wood, 2020).

The profile of the Romanian representatives of Generation Z made by Iorgulescu (2016) indicates that Generation Z does not want to work in isolation but tends to prefer working in groups in open-space offices. Moreover, the study by Iorgulescu confirmed the conclusions of previous research, indicating that Generation Z has a constant need for development, expects to be mentored by its superiors, and desires to develop good working relationships. In addition, the study confirmed that Generation Z has a strong need for security, reflected in their desire for secure jobs and generous pay (Iorgulescu, 2016).

Other studies on Generation Z and education in the university environment show that today’s media-driven generation labeling is not enough to interpret generational characteristics. Törőcsik, together with a group of researchers, states that Generation Z people cannot be uniformly described based on existing research. They do not consist of happy life-starters because they also must struggle with problems. Their confidence and their desire for money and success are typical of them, but they also need help while searching for their identity. The circumstances of this generation are different: for example, they use IT devices, social media, and mobile phones actively (Törőcsik et al., 2014).

Seemiller and Grace (2017) point to the university education of Generation Z in four directions. The first direction refers to teaching-learning strategies, namely the use of video-based learning: Capitalize on Generation Z’s interest in learning through observation by using videos and other visuals to help explain a theory or concept or to demonstrate a challenging process. The second direction refers to incorporating intrapersonal learning into class and group work: Consider breaking a project into multiple “checkpoints” along the way that provide opportunities for individual learning and reflection before having students’ groups complete “checkpoints” later in the process. The third direction refers to offering community engagement opportunities for students to address underlying societal needs, and the fourth is to connect Generation Z students to internship opportunities. Because Generation Z students want their educational experience to incorporate practical learning opportunities from the beginning, they may not want to wait until their later college years to acquire an internship.

Schwieger and Ladwig (2018) state that the characteristics of Gen Z that affect classroom activities include creativity, entrepreneurship, fairness, hands-on experiences, high expectations, multitasking, personalized microexperiences, pragmatism, self-reliance, self-informity, skill-focus, social-media connections, storytelling, trust, and workplace advancement.

Referring to university education in the field of engineering, Moore and Frazier (2017) make the following recommendations regarding the teaching-assessment strategies of Generation Z: integrate active and problem-based learning, help students extract answers from an ocean of information, assess often and provide feedback, engage creativity, and help students make connections.

### 2.4. The aim of the study

Following the analysis of the existing research, we found that studies focusing on Generation Z’s feedback are limited (Sepma et al., 2018). There are many articles in the literature on feedback, but very few on adapting feedback to the needs and expectations of Generation Z.

Currently, at ULBS, the tools used tend to be oriented toward the evaluation of teachers by students on the evaluation criteria of the university partially contained in the job description, and the results are quantified in the form of a grade from 1 to 6. The evaluation tools assess clusters such as content, methods, means, time, communication, applicability, and presence using 21 questions. These questions have a grading scale from 1 to 6, where half of the questions have a maximum score of 6, and the other half have a maximum score of 1. This change results in an 18% student rating error, with students incorrectly marking the maximum option as 6 on all questions.

A feedback questionnaire oriented to the needs of the student has the following benefits:

- Maximizing the potential of each student;
- The ability to diagnosing the needs of each student in terms of physical, cognitive, affective, socio-economic, or cultural characteristics;
- Approaching problems from the student’s perspective;
- Respecting students’ rights and showing an attitude of sensitivity toward their needs and interests (SafeEngine, 2020).

The research aims to develop an innovative feedback tool for the higher education engineering sector to support the improvement of learning outcomes-oriented curricula and teaching activities to better meet the learning needs of students while being relevant to the labor market and to society in general.

A feedback tool built according to the needs and interests of the students improves their level of satisfaction with the teaching process.

Based on the goal of the study, the following research questions arise:

Q1 What are the needs and interests of the students regarding the didactic act?
Q2 How does the level of satisfaction of students regarding the teaching process vary depending on the area of origin?
Q3 To what extent does feedback provided by students change after remedial solutions are applied by teachers?
Q4 Which evaluation tool used at the university level better captures the expectations of students, and which is more effective for the teacher in the sense of providing clear benchmarks for improving the didactic act?

The research questions led to the formulation of the objectives:

Objective 1: Analysis of students’ points of interest regarding the evaluation of teaching staff.
Objective 2: Comparison of the evaluations made by the students according to the environment of origin.
Objective 3: Comparative evaluation of the feedback provided after the application of remedial solutions.
Objective 4: Determination of the optimal homogeneous feedback tool.

The working hypotheses are as follows:

*H1*: Student evaluation of teachers has different points of interest compared to the model provided by the university.

*H2*: Students’ evaluation of teachers varies according to students’ area of origin.

*H3*: Applying remedial solutions leads to giving feedback on the same evaluation matrices.

*H4*: The SKS evaluation form is more reliable in evaluating different disciplines than the standard evaluation form by providing a more homogeneous type of feedback for each discipline or teacher.

## 3. Materials and methods

### 3.1. Overview

The research is part of the project SafeEngine: Blended Learning through Innovative Tools for Sustainable and Safety Engineering and Social Inclusion (ID 2020-1-RO01-KA203-080085), a Strategic Partnership concerned with the development of innovative tools for the engineering higher education sector, supporting the improvement of some learning outcomes-oriented curricula that better meet the learning needs of students while also being relevant to the labor market and the wider society. In collaboration with partners from the technical universities of Bucharest (RO), Malaga (ES), Sibiu (RO), and Naples (IT), the project proposes the development and implementation of four stackable e-learning course modules and related practical works with open online access; the development of best practices, common standards, and guidelines for designing and making e-learning courses; and testing the innovative practices developed in the framework of the SafeEngine project through innovative ICT technologies and mutual learning. The results of the present research will be discussed and analyzed within the general project to be implemented at the level of universities in the three participating countries: Romania, Spain, and Italy.

The present research was carried out based on preliminary research carried out within Lucian Blaga University of Sibiu, Romania, in 2020, which aimed to evaluate the changes produced in the wellbeing of students, the characteristics of the feedback provided on the activity of the teacher, as well as the school results, under the impact of quarantine during the COVID-19 pandemic (Cioca and Bratu, 2021b). A comparison between the current research and previous studies will be made to observe the changes in the longitudinal study.

During the second semester of the 2021–2022 academic year, the research was conducted in the Faculty of Engineering at Lucian Blaga University in Sibiu.

### 3.2. Participants

The research population for this study includes the young people studying at the Faculty of Engineering, Lucian Blaga University of Sibiu, Romania, and teachers working in this institution. The population sampling involved all students who participated in seven teachers’ courses and expressed their free consent, namely 246 students. The seven teachers involved in the research are part of the SafeEngine: Blended Learning through Innovative Tools for Sustainable and Safety Engineering and Social Inclusion project team, described previously.

A total of 18 items were used to describe sample characteristics: 14 describing students and 4 describing teachers. Supplementary Table S1 describes the characteristics of the investigated sample.

The questions describing the sample were chosen to observe if the interregional migration of the population in Romania has an impact on the perception of reality and school activity.

### 3.3. Ethical approval

The study procedure and instruments were approved by the Commission of the Ethics Committee of the Lucian Blaga University of Sibiu, Romania (NO.02-14.07/2022).

A project titled “Generation Z’s Expectations regarding the University Educational Act in Romania: Optimizing the Didactic Process by Providing Feedback” meets the ethical requirements outlined in the code of ethics for scientific research. Before the study, students were informed that participation was voluntary and anonymous. In addition, they were informed about the purpose of the study and about the possibility of resigning from it at any time.

### 3.4. Materials and methods

The feedback request form was created in Google Forms and has three sections. The first section includes questions related to the characteristics of the studied sample. The second section includes the SKS method: STOP (S), KEEP (K), and START (S) (Cioca and Bratu, 2021b). The third section includes the classic teacher evaluation form for students used by ULBS.

#### 3.4.1. SKS method

The SKS method was used and promoted by Professor Philip Daniels (Cioca and Bratu, 2021b) from Brigham Young University, who states that the following three questions are particularly effective for obtaining feedback:

1. What should I give up?
2. What should I continue to do?
3. What should I start doing? (DeLong, 2011).

The SKS method is often used for evaluation within universities and for performance evaluation on Wall Street (DeLong, 2011). Its effectiveness is reflected in the actions that can be taken afterward because it asks for both positive and negative feedback. The feedback tool was used for studies in the fields of education and medicine, where human interaction has a primary role (Sarkany and Deitte, 2017; Sabesan and Whaley, 2018; Cantero-Chinchilla et al., 2020; Tagle et al., 2021). The value of the tool lies in the fact that it describes the personal vision of the evaluator without imposing any limits regarding desirable behaviors. Thus, it can be used to develop a quantitative assessment tool that captures a certain category of people’s interests, expectations, and vision.

Students were asked to answer the three questions freely without any additional cues, allowing them to approach the feedback from any perspective they deemed fit (Cioca and Bratu, 2021b).

To facilitate the interpretation of the data, based on the preliminary research carried out in 2020, four areas of general competencies were defined and built as competence concentration poles, which have as subdivisions 21 areas of professional and transversal competence identified by students, as illustrated in Supplementary Table S2.

The answer to each question was marked with 0 (competence is not mentioned), 1 (positive mention), or 2 (negative mention) on each of the 21 professional and transversal skills, resulting in a total of 63 positions. Both the type of feedback (positive and negative) and the frequency of naming the competencies were analyzed.

The data were interpreted using IBM SPSS Statistics v23.

#### 3.4.2. Classic teacher evaluation questionnaire

The classic teacher evaluation questionnaire includes a total of seven questions and 24 statements, all evaluated on a scale from 0 to 6. Four questions use an increasing scale, with the maximum score on the right side, and three questions use a decreasing scale, with the maximum score on the left side.

### 3.5. Data analysis

To validate our hypotheses 1–4, data were structured in a database. Then, descriptive statistics were used to define and understand how data are constructed. Thus, in the first part of the result section, averages, frequencies, and sums were computed and exposed. After the descriptive statistics were covered, the four hypotheses were analyzed.

To validate hypotheses 2, 3, and 4, different types of t-tests were applied. To determine the equality of the variances between the two samples, the F-test was used.

For validating hypothesis 4, the next data and methods were used, as presented in Supplementary Table S3.

The datasets comprised evaluations from 246 students, assessing 7 teachers across 12 disciplines. This amounted to 14,598 data points for the SKS evaluation form and 5,166 data points for the standard evaluation form.

### 3.6. Research design

**Figure 3:**
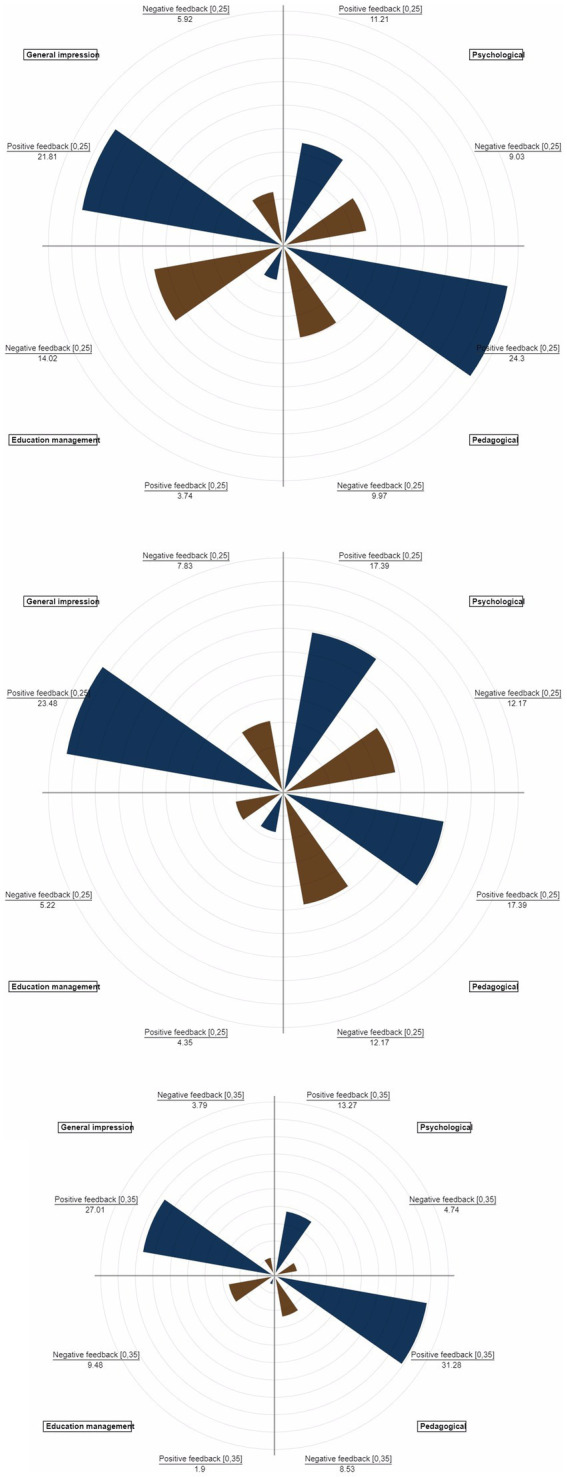
Percentages of areas of competence for teachers 1, 2, and 3.

To easily visualize the obtained data, polar charts were built for the evaluated teachers. Graphs allow visual comparison between the four areas of competence in the form of a two-dimensional chart with positive and negative feedback. At the same time, polar charts allow the identification of each teacher as a unique personality, represented by a circle or a sphere, in which the skills are present in different percentages on the two coordinates, positive and negative. Thus, the possibility of comparing teachers on bar graphs is eliminated. In Figure 3, the graphs display the feedback percentages for three teachers, broken down by areas of competence, relative to the total feedback each teacher received.

## 4. Results

### 4.1. General presentation of the data

**Figure 4:**
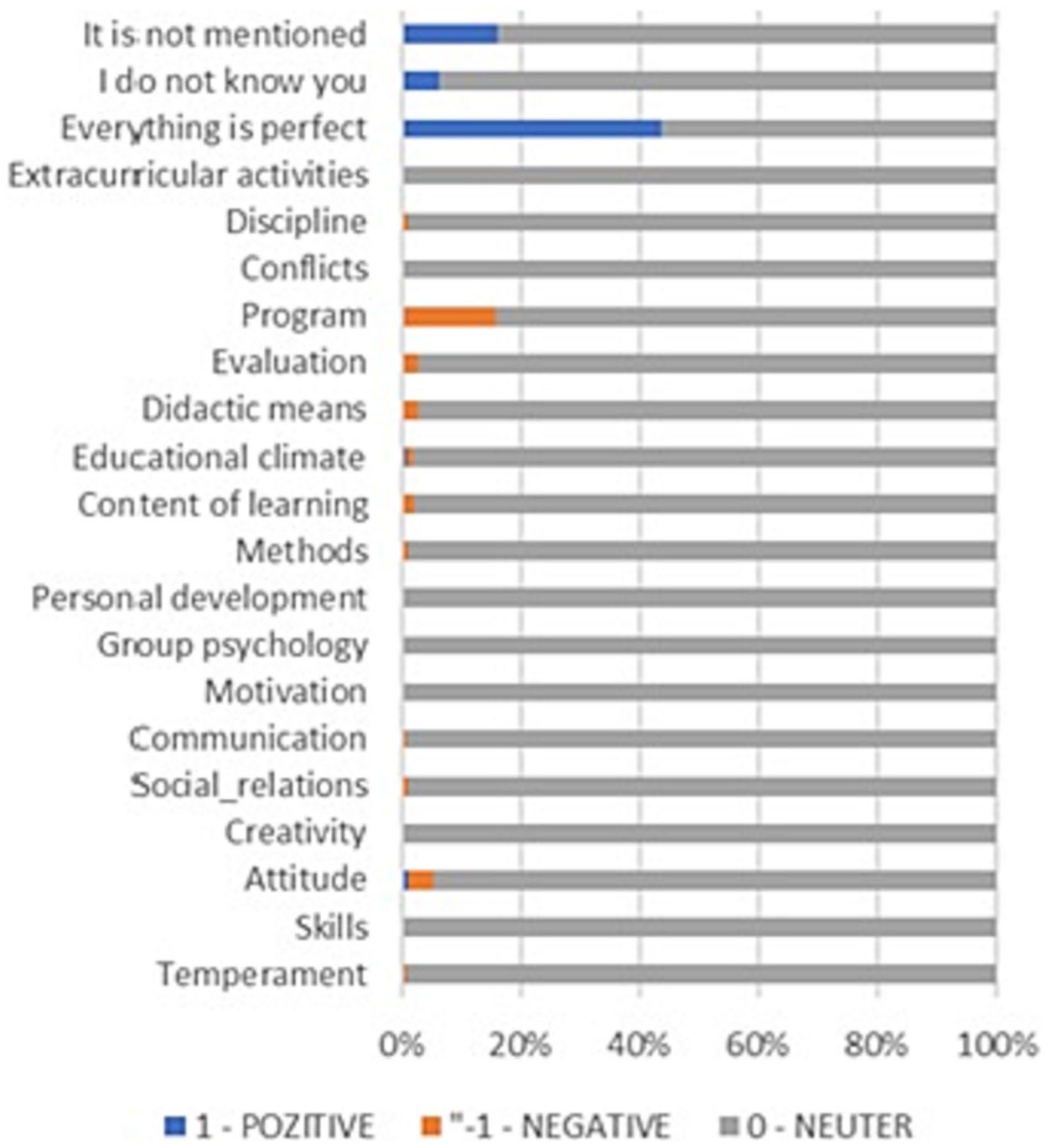
SKS Method. Things to STOP.

**Figure 5:**
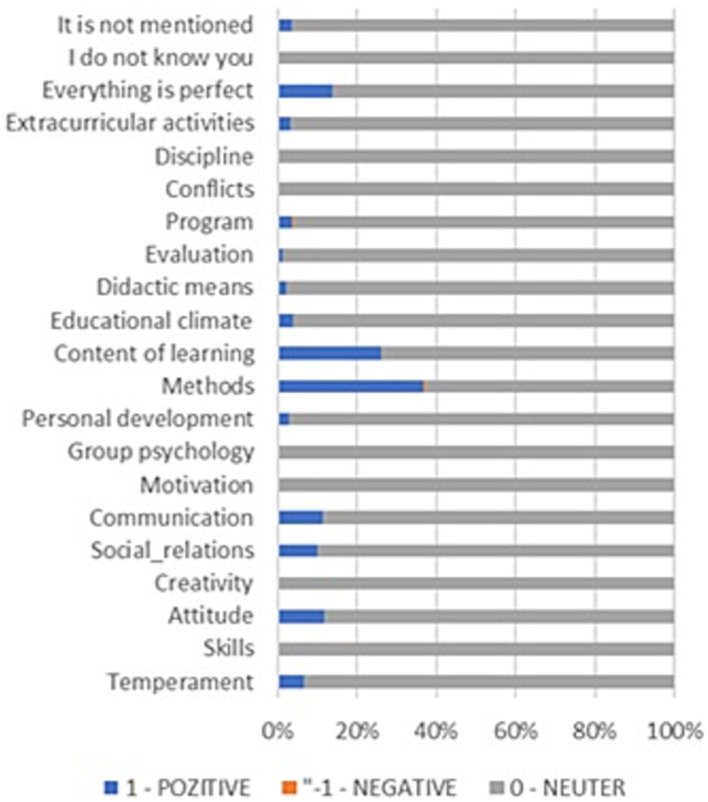
SKS Method. Things to KEEP.

**Figure 6:**
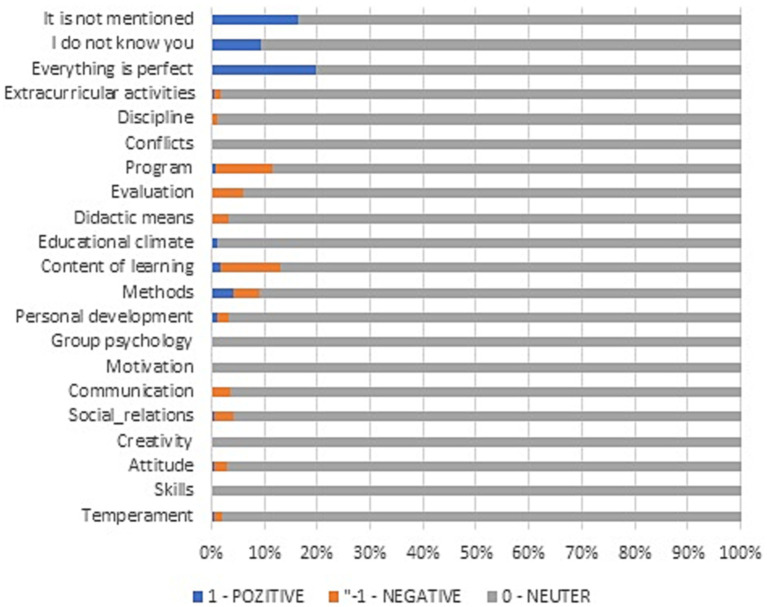
SKS Method. Things to START.

Regarding the SKS evaluation, the frequency of the answers depending on what to KEEP, restart, or STOP is presented in Figures 4–6, from which several conclusions about the data can be drawn. First, aspects that need to be cut out, presented in Figure 4, are mostly negative, but many respondents have responded that everything is perfect. Second, things that need to be kept are mainly positive, as Figure 5 shows, especially regarding methods and content of learning. Third, things that need to be started are mainly negative, especially aspects regarding the program, content of learning, methods, and evaluation.

**Figure 7:**
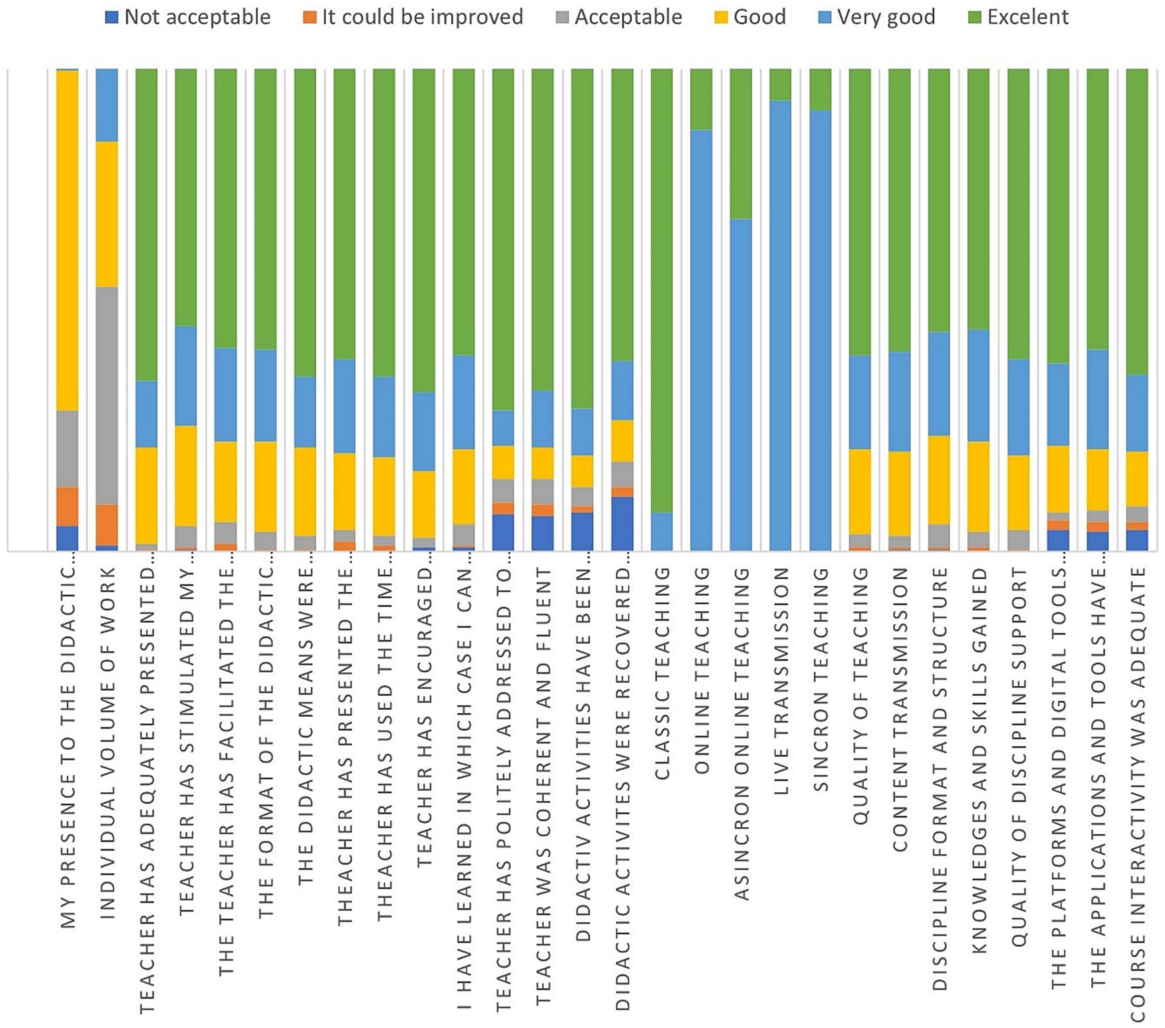
Relative frequency of answers.

In the case of the standard university evaluation form, the relative frequencies of each type of answer (from very bad to excellent) are presented in Figure 7. As one can observe, many students found almost everything to be excellent, except for online teaching in all its three forms (synchrony, asynchrony, and live transmission). Additionally, the individual volume of work is found to be very low or medium.

Total scores on each type of question, in the case of the SKS form, are: STOP 87, KEEP 340, and START 10.

The total scores were computed as follows in Equation 1:


(1) $$\mathrm{Total}\;\mathrm{scor}e_{x}=\sum \mathrm{Each}\;\mathrm{individual}\;\mathrm{score}\;for\;\mathrm{each}\;\mathrm{question}\;\mathrm{type}\;X$$

where X = STOP, KEEP or START.

### 4.2. Average scores for each type of data

**Figure 8:**
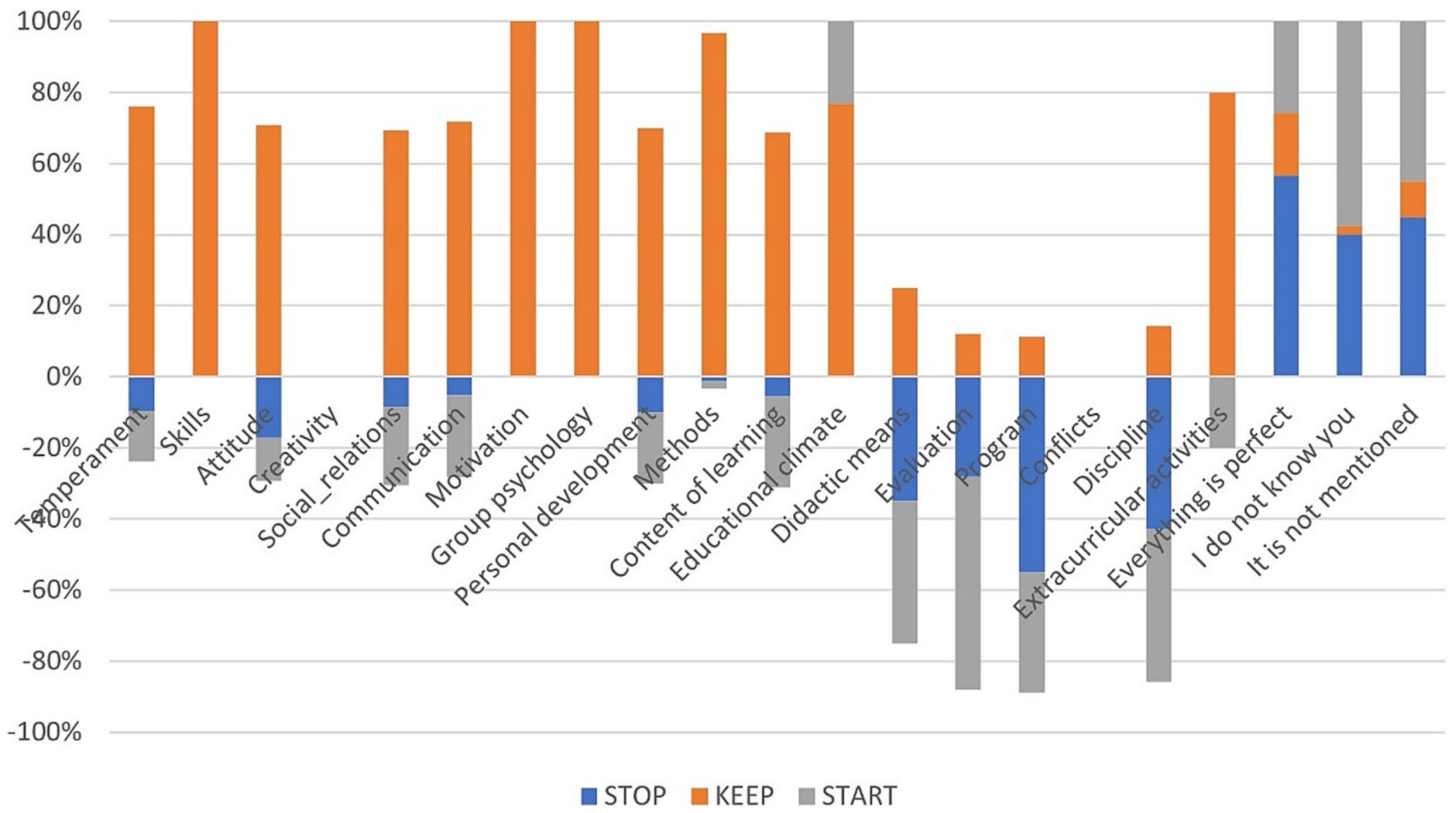
Average scores for each type of skill for SKS evaluation.

**Figure 9:**
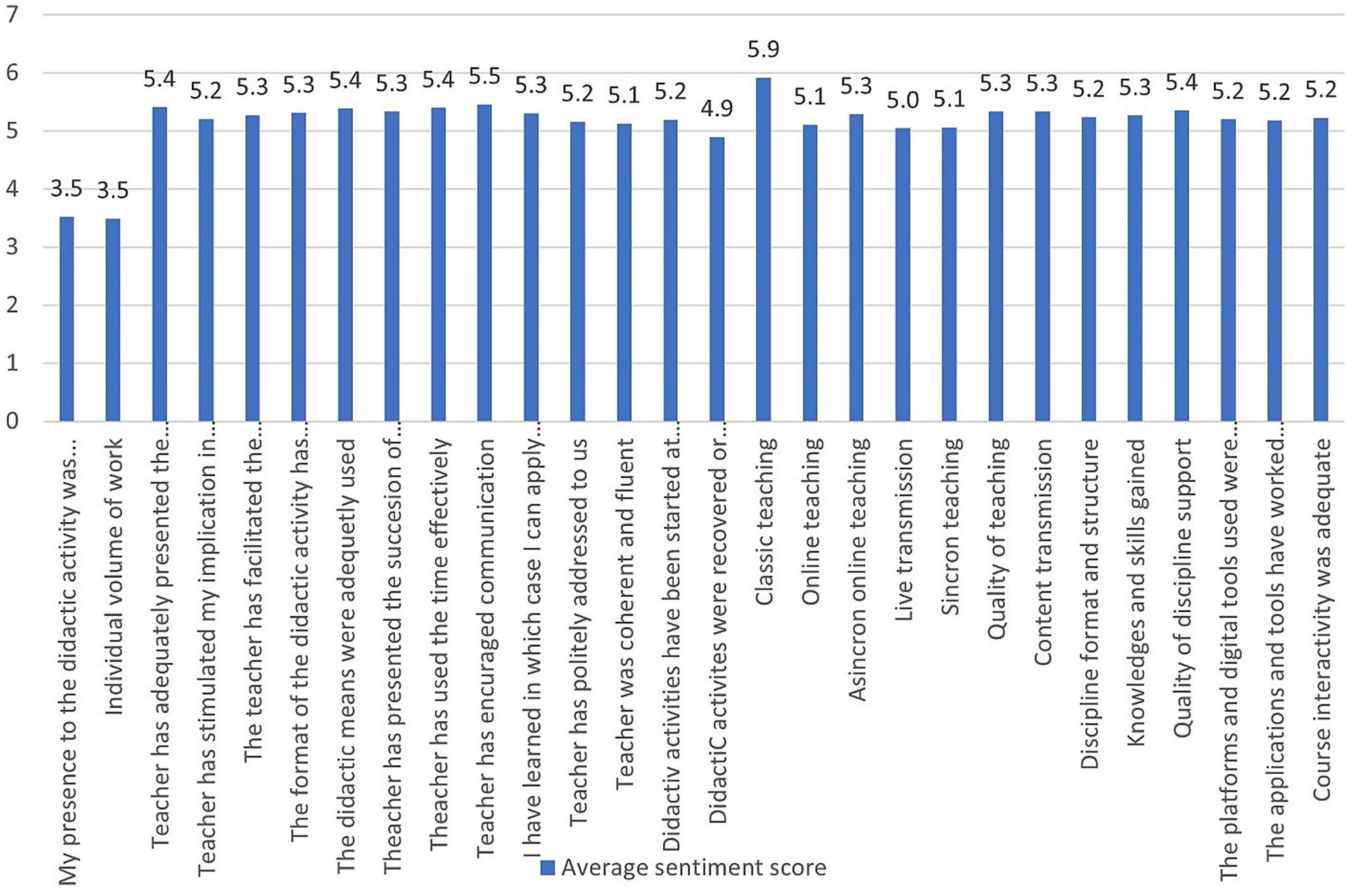
Average sentiment score for each dimension for standard evaluation.

In Figure 8, it can be found that, in general, things that need to be kept had an average score with a positive sign, while things that need to be cut out or started had mostly negative signs in the case of the SKS evaluation. Moreover, in the case of the standard evaluation, in Figure 9, the average for each question is somewhere above medium, so no meaningful insights can be drawn from this type of indicator in the case of the standard evaluation in comparison with the SKS, where it can be clearly observed that, in the case of didactic means, evaluation, program, and discipline can be applied to some remedial solutions.

AVERAGE scores for each question were computed as follows from Equation 2.


(2) $$\mathrm{Average}\;\mathrm{scor}e_{Q}=\frac{\sum \mathrm{Each}\;\mathrm{individual}\;\mathrm{score}\;for\;\mathrm{each}\;\mathrm{question}}{N}\text{,}$$

where Q represents each question, and N represents the number of respondents (246).

In the case of the averages of individual answers for both evaluation forms, they were computed using Equation 3:


(3) $$\begin{array}{l} \mathrm{Average}\;\mathrm{individual}\;\mathrm{score}s_{i}\\ \;=\frac{\sum \mathrm{Each}\;\mathrm{individual}\;\mathrm{score}\;for\;\mathrm{each}\;\mathrm{question}\;for\;\mathrm{the}\;\mathrm{subject}\;i\;}{n}\text{,} \end{array}$$

where *i* represents the subject and *n* represents the number of questions in each form.

**Figure 10:**
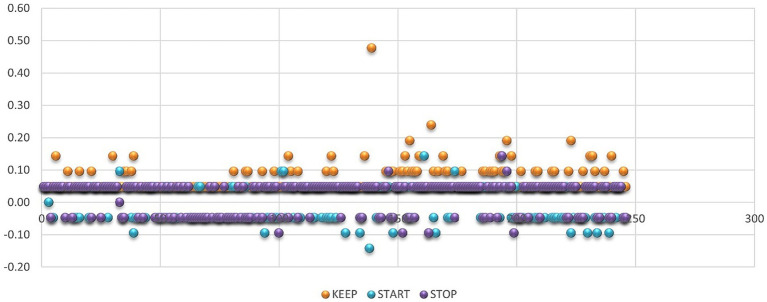
Average of individual scores – SKS.

**Figure 11:**
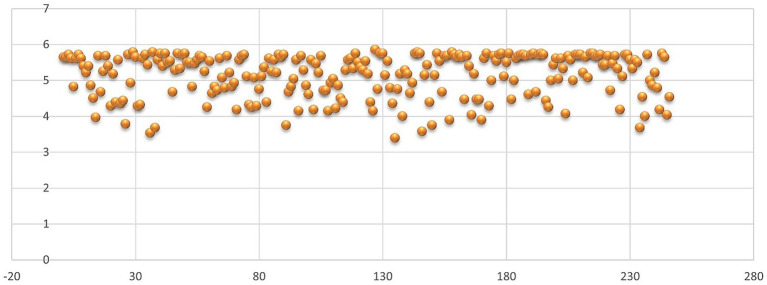
Average of individual scores – standard form.

It can be seen from Figure 10 that, in the case of SKS, the individual responses are mainly positive, with the most positive responses to things that need to be kept and the most negative individual scores for things that need to be restarted, while in the case of the standard evaluation, presented in Figure 11, the scores are mostly above the medium level of 3.5.

### 4.3. Objective 1: analysis of students’ points of interest regarding the evaluation of teaching staff

#### 4.3.1. Validating hypothesis 1

*H1*: The evaluation of teachers by students has different points of interest compared to the model offered by the university.

To observe the points of interest in the two forms, it is necessary to analyze the related questions in the two evaluation tools.

The validation of the first hypothesis is based on the correlations presented in Supplementary Table S4, along with the examination of supplementary factors of interest, including temperament, attitude, creativity, personal development, and other relevant aspects. Upon analyzing Supplementary Table S1, it becomes evident that measurements related to communication, adequate use of methods, administration time, and the format and structure of the discipline demonstrate consistent alignment with both instruments. However, the remaining measurements offer only partial or inconsequential information regarding the didactic performance, particularly concerning the cluster of teachers and university resources.

### 4.4. Objective 2: comparison of the evaluations made by the students according to their environment of origin

#### 4.4.1. Validating hypothesis 2

*H2*: Students’ evaluation of teachers varies according to students’ area of origin.

Correlations among demographic variables and average individual scores for STOP, START, and KEEP sentiments.

Hypothesis 2 is explained by the Pearson correlation coefficient, presented in Supplementary Table S5, as it is very significant in the area of provenience and the individual score for teacher evaluation. Thus, the correlation between the aspects that should be kept in the didactic activity and the type of area of provenience is negative, with a 0.095 level of significance. A higher level of significance is attributed to the correlation between aspects that need to be started in economic activities and the type of area of provenience (rural/urban), still maintaining a negative relationship. In addition, in relation to the country areas, there is a very significant correlation, with a sig. of 0.000, with the things that should be kept in the didactic activity.

To perform the two-sample t-test, we had to compute the F-test to ascertain whether the variances for the three types of scores, stop, keep, and start, were equal or unequal. The results of the F-test are shown in Supplementary Table S6.

As was mentioned in the case of the start and stop individual scores, the variances are considered unequal. However, the variance is found to be equal; thus, for the first two mentioned cases, we have used the t-test: Two-Sample Assuming Unequal Variances, while for the “keep” score, we have used the t-Test: Two-Sample Assuming Equal Variances. The results are shown in Supplementary Table S7.

**Figure 12:**
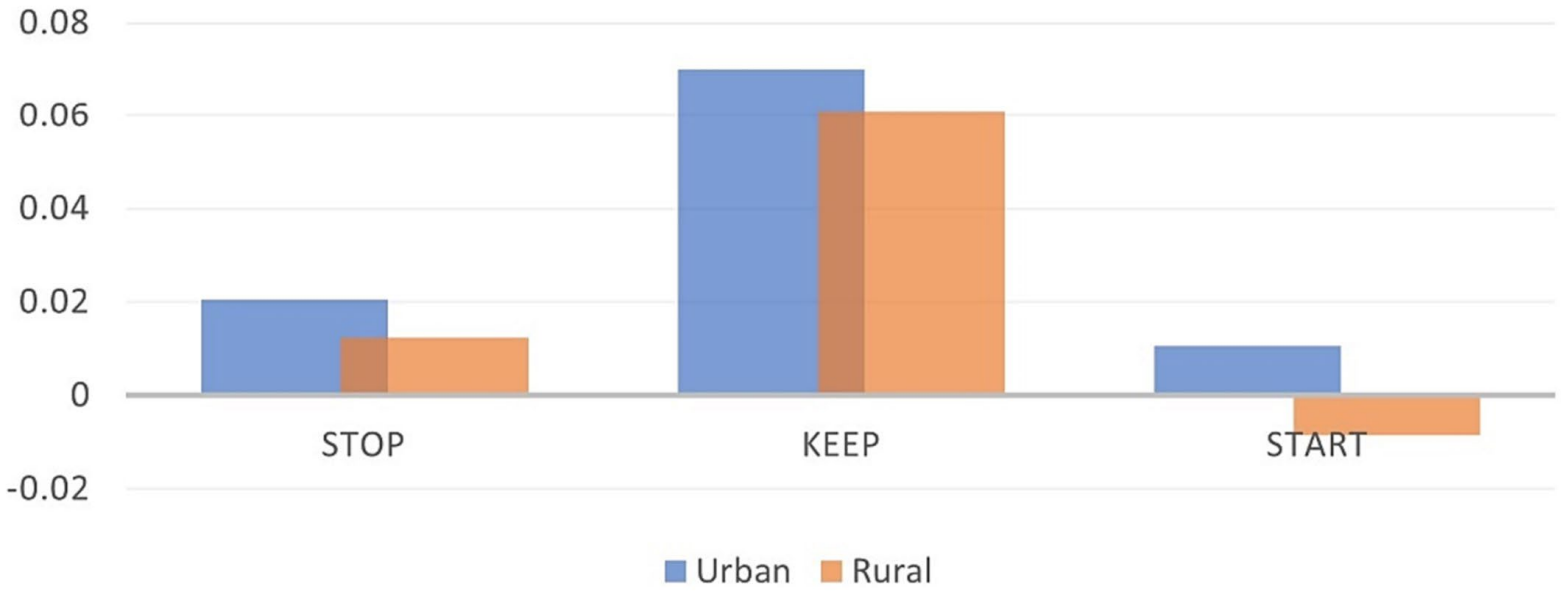
Averages of individual scores – Urban and rural.

**Figure 13:**
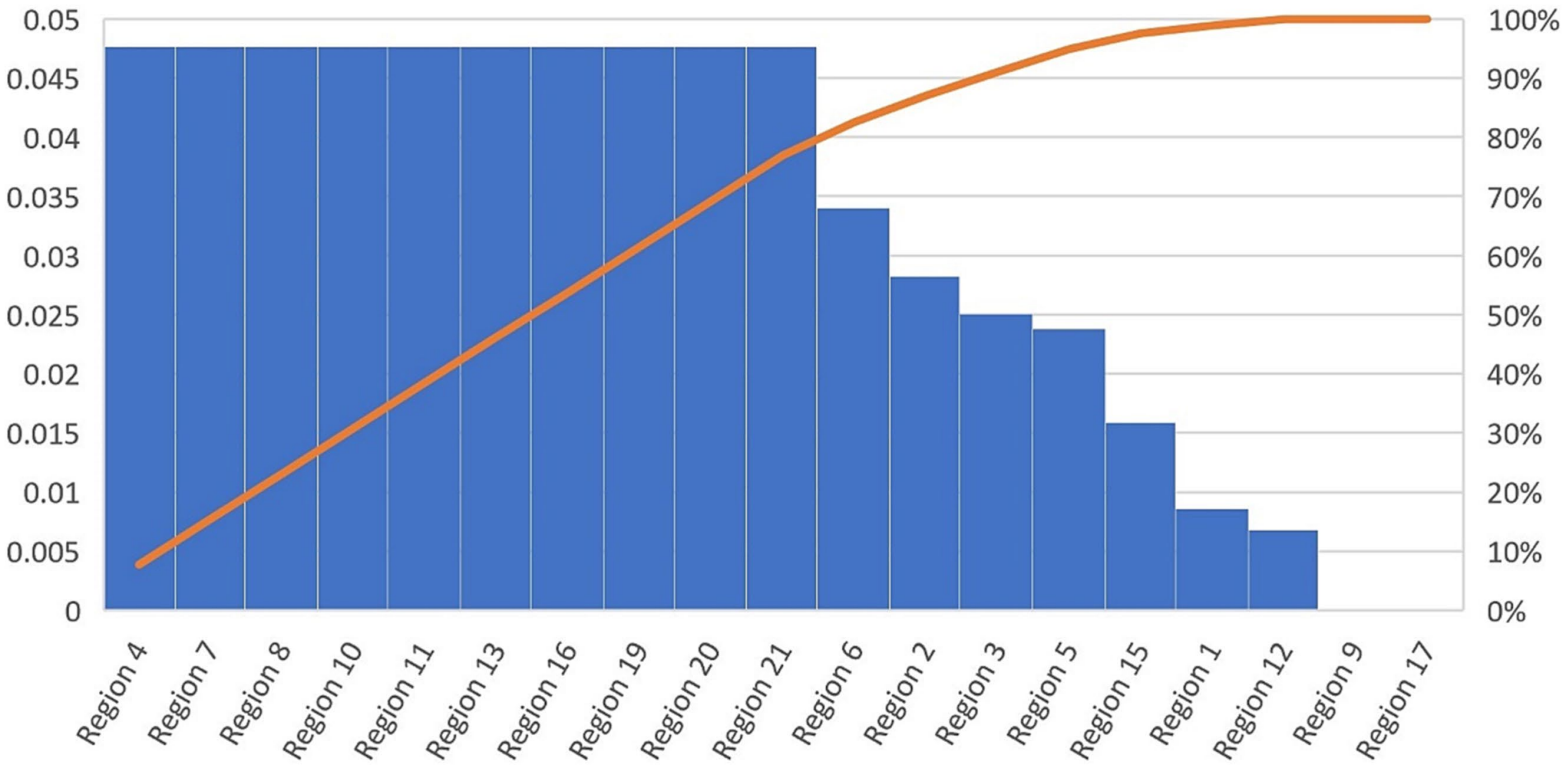
Correlations among questions and demographic variables.

Using the value of p from Supplementary Table S7, the null hypothesis that there is no significant difference between the means of the individual scores from two different groups (rural and urban provenience groups) is rejected in the case of the aspects that need to be started or restarted, with a significance of 0.01, and in the case of the things that need to be kept in the didactic activities, with a significance of 0.1 (Figures 12, 13).

**Figure 14:**
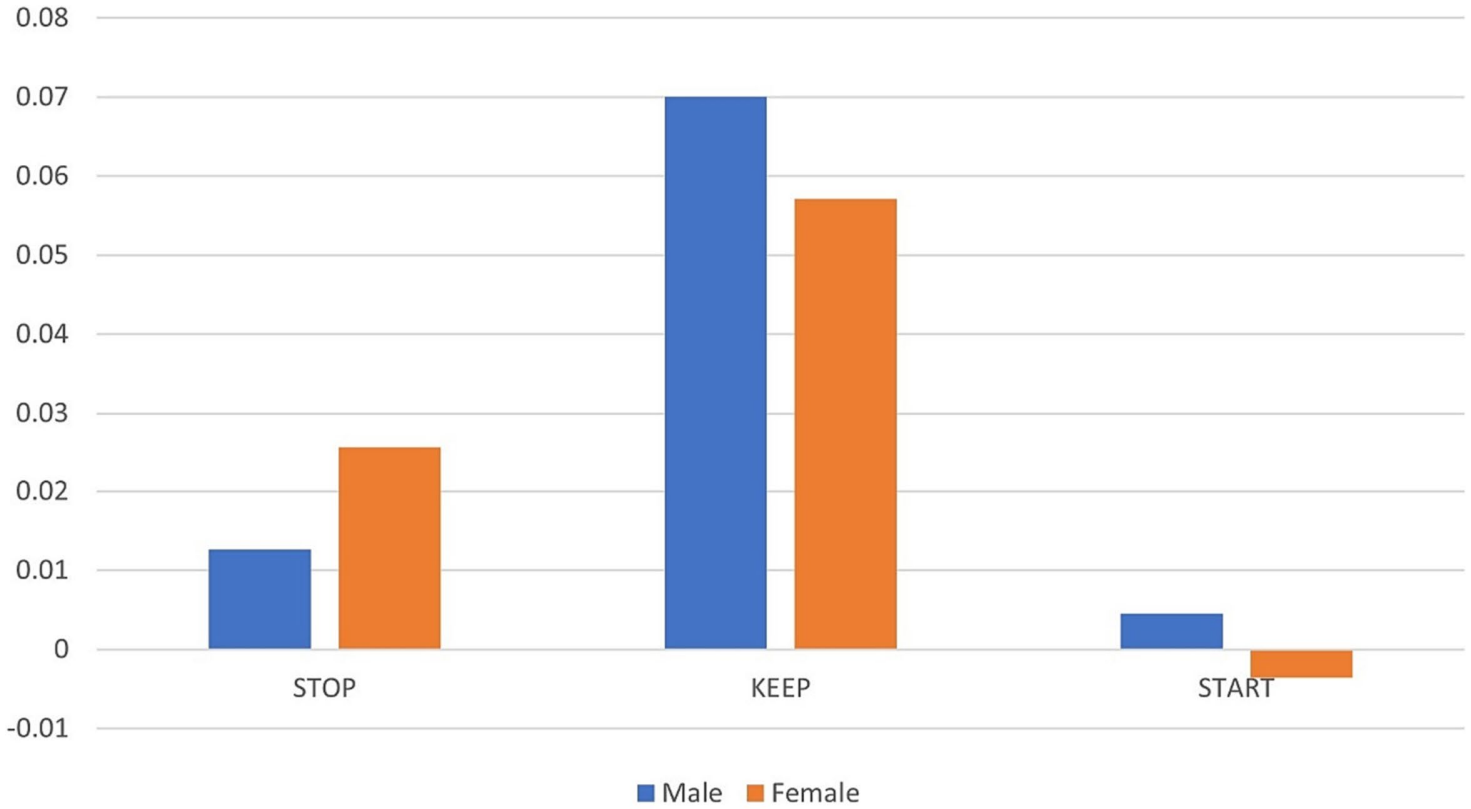
Averages of individual scores.

As observed in Supplementary Table S8, correlations among gender and pedagogical aspects to be kept are significant and positive, while they are negative among aspects that need to be stopped. In this case, women, denoted with the 2-dummy variable, are more likely to respond negatively to things that need to be started, while men are more likely to respond positively (Figure 14).

Furthermore, in the case of age, a significant, positive correlation is found between strings that need to be cut out and the subject’s age.

While the correlation coefficient among individual scores and living with the parents is found to be significant and positive, when talking about aspects that need to be cut out, with a sig. Value under 0.05, correlations with types of net income are insignificant, stop-living with parents 0.155*.

### 4.5. Objective 3: comparative evaluation of the feedback provided after the application of remedial solutions

#### 4.5.1. Validating hypothesis 3

*H3*: The application of remedial solutions results in feedback based on the same evaluation matrices.

**Figure 15:**
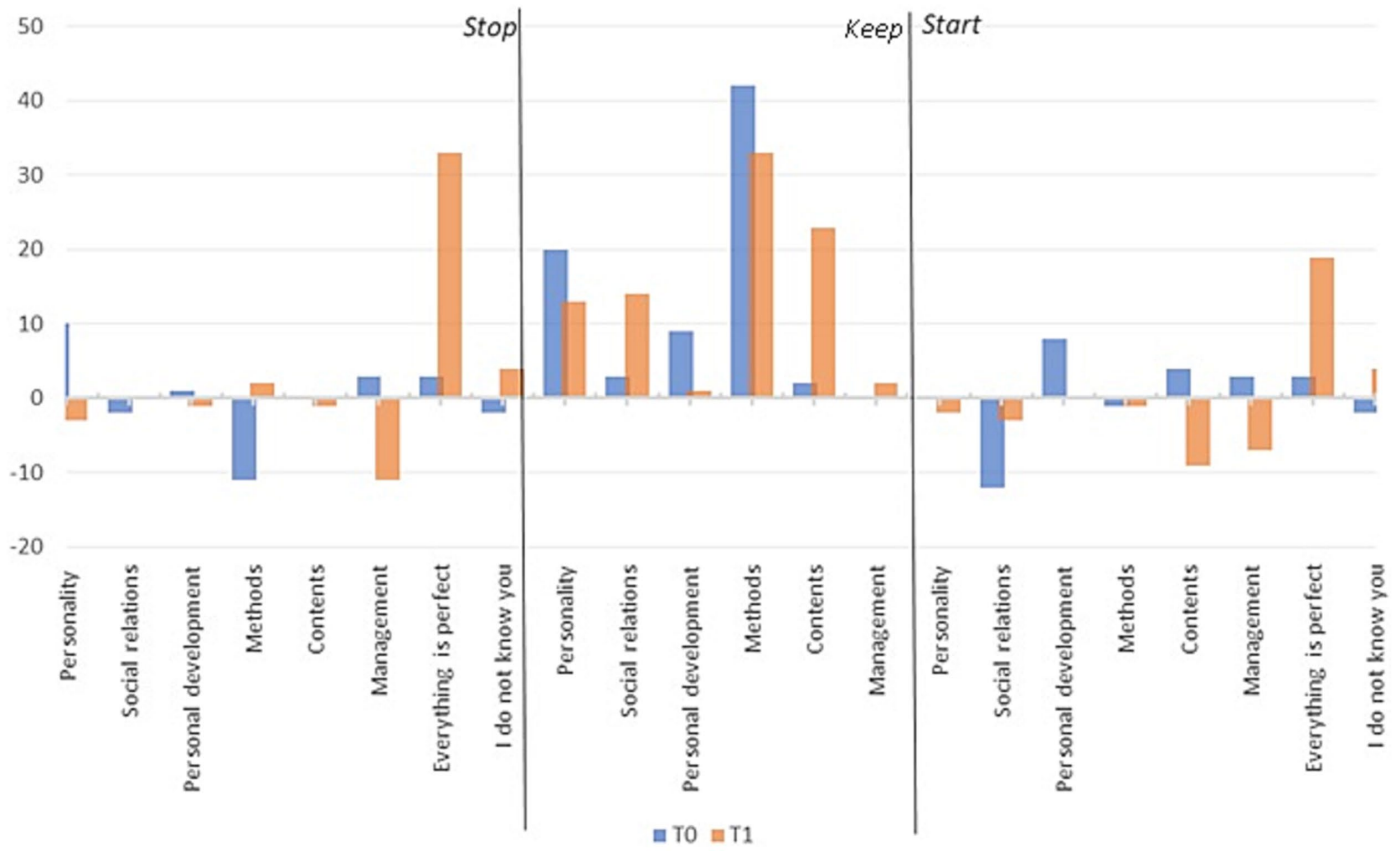
Average of individual scores T0 – T1.

To validate H3, we have made use of an old set of data from a previous year, which we will compare with the actual set of data for discipline 10 and teacher 5. In the period between the application of the two forms and the collection of the two sets, the teacher has provided solutions for the problems found in the first set of data. We will then observe if the remedial solutions have led to feedback offerings on the same evaluation matrices as the first dataset. Thus, in Figure 15, it can be observed that from T0 to T1, aspects such as the perception of perfection, wholesome methods, and social relations no longer need to be included; they have been present based on the remedial solutions; thus, these aspects should be retained since they are mostly present.

To validate the efficacy of the remedial methods taken, a t-test has to be run. Thus, an F-test is used to determine if the two datasets have different variances, as shown in Supplementary Table S9. From Supplementary Table S8, we can determine that the two variances differ. Thus, the t-Test: Two-Sample Assuming Unequal Variances will be applied.

The results, presented in Supplementary Table S10, show that the remedial solutions had no impact on the secondary dataset. Thus, different remedial solutions need to be taken. Although we analyze the dataset in detail, we can observe that the overall perspective that everything is perfect has improved in the analyzed period.

### 4.6. Objective 4: determination of the optimal homogeneous feedback tool

#### 4.6.1. Validating hypothesis 4

*H4*: The SKS evaluation form is more reliable than the standard evaluation form when evaluating different disciplines. This is because it provides more homogenous feedback for each discipline or teacher.

As can be seen from Supplementary Tables S11, S12, the SKS evaluation form provides significant correlations for three to five variables (depending on the attribute type: KEEP, STOP, or START action) in relation to the evaluated teacher or discipline. In contrast, the standard evaluation form only shows significant correlations for two variables. Notably, the students’ presence in the activity is not an evaluation variable. This is out of a total of 21 and 20 variables for both forms, respectively. Thus, hypothesis 4 is validated first by the number of significant correlations that the SKS form provides among the different variables with the disciplines and teachers, thus providing a homogenous type of feedback (positive, negative, or neutral) for disciplines and teachers. Second, the significant. values for the correlations found in the case of the SKS form are lower than the ones provided for the standard form. Thus, the scores and feedback provided for each discipline and teacher are more trustworthy in the SKS form than the standard one.

## 5. Discussion and conclusion

In the present study, we aim to analyze a new tool for evaluating the didactic activity in superior education for 12 economic and technical disciplines. The study involved 246 subjects, all of whom were students with 1–4 years of undergraduate experience and an additional 2 years of master’s study. Moreover, the present evaluation tool aims to analyze both the efficiency of the seven teachers’ activities and the usefulness of the resources put at their disposal by the university. To analyze the performance and efficiency of the tool, it was compared to the standard evaluation tool proposed by the university for evaluating the didactic activity. The results were compared in terms of means, sums, average individual scores per question or subjects, t-tests, and bivariate Pearson’s correlations. Four hypotheses were tested using the results of the present article:

*H1*: Student evaluation of teachers has different points of interest compared to the model provided by the university.

*H2*: Students’ evaluation of teachers varies according to students’ area of origin.

*H3*: Applying remedial solutions leads to giving feedback on the same evaluation matrices.

*H4*: The SKS evaluation form is more reliable in evaluating different disciplines than the standard evaluation form by providing a more homogeneous type of feedback for each discipline or teacher.

The results show that the first hypothesis is validated by the significant correlations presented in Supplementary Table S2 among the related questions from the two evaluation forms and by the existence of the supplementary point of interest in the SKS evaluation method. Students’ evaluations of teaching effectiveness pose a serious challenge to existing programs that assume that feedback is sufficient to improve teaching effectiveness (Marsh, 2007). The more the students’ interests diverge from those of the teachers, the less effective the feedback is in improving the didactic act.

To analyze hypothesis 2, the t-test was applied, concluding that there is a significant difference between the means of the individual scores from two different groups (rural and urban provenience groups) in all three cases: STOP, START, and KEEP.

Regarding hypothesis 3, the results show that the remedial solutions had no impact on the secondary dataset; thus, different remedial solutions need to be taken, although if we analyze the data in detail, we can observe that the overall perspective that everything is perfect has improved through the analyzed period. The study by Yarbro and colleagues confirmed that the model contributed to the enrichment of teaching and learning processes, aspects related to the integration of concepts, the role change of professors and students, the improvement of the processes of participation and communication, the improvement of academic results, and the promotion of student interest in the course (Flores et al., 2016).

Hypothesis 4 is validated first by the number of significant correlations that the SKS form provides among the different variables with the disciplines and teachers, thus providing a homogenous type of feedback (positive, negative, or neutral) for disciplines and teachers. Second, the Sig. values for the correlations found in the case of the SKS form are lower than the ones provided for the standard form. Thus, the scores and feedback provided for each discipline and teacher are more trustworthy in the SKS form than in the standard one (Sabesan and Whaley, 2018; Cantero-Chinchilla et al., 2020; Tagle et al., 2021).

The main advantage of using the SKS method instead of the standard method is the full view of the students’ perceptions, as evident from the results, where average scores, both per subject and per question, are more heterogenous for the SKS model, while the correlation of the questions with the evaluated discipline is higher, thus enforcing much more significant responses that could define the students’ actual needs and priorities. Moreover, the actuality of the questions in correspondence with the macroeconomic context is more suitable and flexible in the case of the SKS form, as the standard evaluation includes questions related to online activity or types of activity structure in the case of online courses.

The ease of identifying remedial actions is more effective in the SKS model, as it allows free answers from three perspectives (things that need to be stopped, included, or kept), as the main purpose of the SKS model is to find remedial solutions. Regarding the pedagogical needs of the students, both the SKS model and the standard model provide scores for the program, discipline, methods, means, and content. Supplementary needs covered by SKS include conflicts, educational climate, and extracurricular activities, while the most novel part of the SKS model stands in the personality’s identification skill remedial solutions that are included in the first part, such as temperament, skills, attitude, and creativity.

In pedagogical literature, feedback is a method that has an impact on the motivation, activity, problem-solving skills, and games of students. Generation Z expects active teaching methods, continual feedback, creative access, and meaningful connections in teaching-assessment strategies (Moore and Frazier, 2017).

Following the application of the feedback questionnaires and the interpretation of the 19,764 data points, we identified Generation Z’s expectations. These revolve around interactive methods and engaging content. Following that, communication, attitude, and social relations are also emphasized. The program and evaluation register the highest number of negative feedback.

For teachers, the option of free feedback offered by students on the three SKS dimensions is proposed as an opportunity for reflection and introspection and as a challenge for change. At the same time, it is recommended that the individual results be represented graphically in the form of polar charts in the four areas of competence, with positive and negative values. Such a representation allows teachers to quickly interpret the data and compare it with personal profiles from previous years.

An aspect that is highly important for the management of the faculty or university is the identification of the material resources that need to be put at the student’s disposal in the didactic activity, which can be more easily determined using the SKS model, which allows free answers.

As a result of the research carried out, the following practical implications regarding the provision of feedback by students regarding the activity of teaching staff can be drawn, which can be useful to university professors, psychologists, and social workers:

- The use of a descriptive feedback tool leads to a variety of behaviors in the direction of teacher evaluation.
- Starting from descriptive instruments, quantitative instruments can be developed (demersal carried out in research), which would summarize the behaviors expected by the current generation by fields of competence.
- The assessment tools should be as simple as possible, with clear assessment scales that do not create errors in the assessment.
- Data analysis should be centralized, and their interpretation should be done visually, through radar-type graphs that provide a unified perspective on the teacher’s personality type, to avoid progress charts that can have positive/negative connotations and that can increase teachers’ resistance to change. They should be organized by areas of competence, which encompass several types of observed behaviors.
- To encourage the feedback process to evolve as something natural and a *sine qua non*-condition.

## 6. Limitations

The following limitations should be considered when generalizing our findings. First, this study used self-reported instruments. Thus, the results may reflect either an overestimation or an underestimation of these conditions. However, subjective measures, while reliable and valuable, are often used in the literature to estimate tested variables. Second, the tool should be tested on a larger number of students from different faculties before implementation.

The main disadvantage of the SKS model is related to the higher amount of time required to complete the evaluation form and the need for more time to cluster and interpret the answers.

Future studies may focus on applying the model to different types of disciplines and comparing the results with the present ones. At the same time, it is extremely important that the presentation of the results of such questionnaires given to students be done in an accessible graphic form that directly presents the areas of competence along with the strengths and future directions of development of the teachers. At this moment, evaluations are collated into graphs that detail each analyzed aspect for each item, making it difficult for teachers to follow. The style of presentation fails to provide a comprehensive vision. Therefore, we emphasize the crucial need for both suitable evaluation tools and graphic representations sorted by competence areas for final results, an aspect that we want to elaborate on in future studies.

## Data availability statement

The datasets presented in this study can be found in online repositories. The names of the repository/repositories and accession number(s) can be found at: doi: 10.4121/21878148.v1.

## Ethics statement

The studies involving humans were approved by Commission of the Ethics Committee of the Lucian Blaga University of Sibiu, Romania (NO.02-14.07/2022). The studies were conducted in accordance with the local legislation and institutional requirements. The participants provided their written informed consent to participate in this study.

## Author contributions

All authors listed have made a substantial, direct, and intellectual contribution to the work and approved it for publication.
