# Supplementary material for: The expectations of generation Z regarding the university educational act in Romania: optimizing the didactic process by providing feedback
Source: Front Psychol. 2023 Sep 29;14:1160046. doi: 10.3389/fpsyg.2023.1160046 (PMC10572363; doi:10.3389/fpsyg.2023.1160046)
Supplement: Supplementary file 1 [file Table_1.docx]

**Table 1.** Sample structure.

| Students sample structure | | |
| --- | --- | --- |
| Personal data | 1. Field of study | Bachelor's degree 84%  Master's degree 16% |
|  | 2. Year of study | Bachelor 1-2 12%  Bachelor 3-4 73%  Master 1-2 15% |
|  | 3. Gen | Female 67.5 %  Male 32.5 % |
|  | 4. Age | 19-25 years 83%  26-30 years 11%  Over 30 years 6% |
| Demographic data | 5. Study in your hometown | Yes 38%  No 62% |
|  | 6. The environment of origin | Urban 54.5%  Rural 45.5% |
|  | 7. Native region | Center 65.8%  Northeast 0.4%  West 1.2%  Northwest 0.8%  Southeast 1.6%  South-Mountain 1.2%  Bucharest-Ilfov 2%  South-West Oltenia 23.6%  Other country 3.4% |
|  | 8. I live with my parents | Yes 48%  No 52% |
|  | 9. I have lived without my parents since the age of… | 15 years 5.7%  18 years 37.4%  23 years 6.9%  Not the case 50% |
|  | 10. I visit my parents' house | Weekly 27.2%  Monthly 16.8%  Annual/ occasional 8%  Not the case 48% |
|  | 11. What is the main reason you left home | Studies 30%  Autonomy 10%  Service 4%  Marriage 3%  Standard of living 3%  Not the case 48% |
|  | 12. You think it was a good decision to move away from your parents | Not the case 48%  Yes 50%  Not 2% |
|  | 13. What were the problems you faced when you left home | Not the case 68%  Finance 8%  Loneliness 5%  Adaptation 5%  Parents 5%  To fend for myself 4%  Other problems 5% |
|  | 14. Average personal monthly income | Under 2000 lei 20%  Between 2001 – 4000 lei 49%  Between 4001 – 6000 lei 20%  Over 6001 lei 11% |
| Teachers sample structure | | |
| Data on the discipline taught | 15. Discipline taught | Technique 5%  Economy 95% |
| Personal data | 16. Gen | Female 53.3%  Male 46.7% |
|  | 17. Age | Under 30 12%  31 – 40 years 0%  41 – 50 years old 44%  51 – 60 years 44% |
|  | 18. Teaching experience | Under 5 years 12%  6 – 10 years 0%  11 – 20 years 18%  21 – 25 years 27%  Over 25 years 43% |
