# Supplementary material for: The expectations of generation Z regarding the university educational act in Romania: optimizing the didactic process by providing feedback
Source: Front Psychol. 2023 Sep 29;14:1160046. doi: 10.3389/fpsyg.2023.1160046 (PMC10572363; doi:10.3389/fpsyg.2023.1160046)
Supplement: Supplementary file 2 [file Table_2.docx]

**Table 2.** Feedback interpretation clusters.

| **No.** | **Areas of competence** | **Professional and transversal competences** |
| --- | --- | --- |
| 1. | Psychological | Personality – Temperament |
|  |  | Personality – Skills |
|  |  | Personality – Character – Attitude |
|  |  | Personality – Creativity |
|  |  | Intersocial relations – Social relations |
|  |  | Intersocial relations – Communication |
|  |  | Intersocial relations – Motivation |
|  |  | Intersocial relations – Group psychology |
|  |  | Personal development |
| 2. | Pedagogical | Methods |
|  |  | Content of learning |
|  |  | Educational climate |
|  |  | Didactic means |
|  |  | Evaluation |
| 3. | Education management | Program |
|  |  | Conflicts |
|  |  | Discipline |
|  |  | Extracurricular activities |
| 4. | General impression | Everything is perfect |
|  |  | I do not know you |
|  |  | It is not mentioned |
