# Supplementary material for: The expectations of generation Z regarding the university educational act in Romania: optimizing the didactic process by providing feedback
Source: Front Psychol. 2023 Sep 29;14:1160046. doi: 10.3389/fpsyg.2023.1160046 (PMC10572363; doi:10.3389/fpsyg.2023.1160046)
Supplement: Supplementary file 3 [file Table_3.docx]

**Table 3.** Data and methods used to validate the hypothesis.

| Hypothesis | Sample 1 | Sample 2 | Method | Validated |
| --- | --- | --- | --- | --- |
| 1 | SKS -t1 | Standard -t1 | Pearson correlations | Yes |
| 2 | SKS -t1 -rural | SKS- t1 -urban | T- test: two samples with equal and unequal variances | Yes |
| 3 | SKS -t0 | SKS -t1* | T- test: two samples with unequal variances | Yes |
| 4 | SKS -t1 | Standard -t1 | Pearson correlations | Yes |
