# Supplementary material for: The expectations of generation Z regarding the university educational act in Romania: optimizing the didactic process by providing feedback
Source: Front Psychol. 2023 Sep 29;14:1160046. doi: 10.3389/fpsyg.2023.1160046 (PMC10572363; doi:10.3389/fpsyg.2023.1160046)
Supplement: Supplementary file 4 [file Table_4.docx]

**Table 4.** Theoretically related questions

|  | Theoretically related questions: | | |  |  |
| --- | --- | --- | --- | --- | --- |
|  | SKS | AVG SCORE^a^ | Standard | AVG SCORE^b^ | Correl^c^ |
| 1 | Skills | 0.33 | Knowledges and skills gained | 5.26 | - |
| 2 | Socialrelations | 4.67 | Course interactivity was adequate | 5.23 | - |
| 3 | Communication | 5.67 | Teacher has encouraged communication | 5.46 | .148^*^ |
| 4 | Motivation | 0.33 | Teacher has stimulated my implication in studying the disciplines curriculum | 5.21 | - |
| 5 | Group psychology | 0.33 | Teacher was coherent and fluent | 5.13 | - |
| 6 | Methods | 28.7 | Individual volume of work | 3.49 | - |
|  |  |  | Teacher has adequately presented the content of the discipline | 5.41 | .138^*^ |
|  |  |  | The format of the didactic activity has facilitated the understanding of the concepts | 5.31 | - |
| 7 | Content of learning | 11.7 | Content transmission | 5.33 | - |
| 8 | Educational climate | 4.33 | Teacher has used the time effectively | 5.4 | .136^* (KEEP)^  and  .140^* (STOP)^ |
| 9 | Didactic means | -3.33 | The didactic means were adequately used | 5.39 | -.125 |
|  |  |  | Quality of discipline support | 5.35 | - |
|  |  |  | The platforms and digital tools used were helpful | 5.2 | - |
|  |  |  | The applications and tools have worked properly | 5.18 | - |
| 10 | Evaluation | -6.33 | Teacher has presented the succession of the housework | 5.33 | - |
| 11 | Program | -18.3 | Didactic activities have been started at the appointed hour | 5.2 | - |
|  |  |  | Didactic activities were recovered or replaced with online activities | 4.9 | - |
| 12 | Conflicts | 0 | Teacher has politely addressed to us | 5.16 | N/A |
| 13 | Discipline | -1.67 | The teacher has facilitated the interdisciplinary connection | 5.27 | - |
|  |  |  | Discipline format and structure | 5.24 | -.210^**^ |
| 14 | Extracurricular activities | 2 | I have learned in which case I can apply the learned concepts | 5.3 | - |
|  | ^a^Max=247,min=-247 | | |  |  |
|  | ^b^Max=6 ,min=1 | |  |  |  |
|  | ^c^N/A = Not applicable, correlation cannot be computed because at least one of the variables is constant.  **. Correlation is significant at the 0.01 level (2-tailed).  *. Correlation is significant at the 0.05 level (2-tailed). In the rest of the cases, correlation is significant at the 0.1 level (2-tailed). Where is present, no significant correlation was found. | | | | |
