# Supplementary material for: The expectations of generation Z regarding the university educational act in Romania: optimizing the didactic process by providing feedback
Source: Front Psychol. 2023 Sep 29;14:1160046. doi: 10.3389/fpsyg.2023.1160046 (PMC10572363; doi:10.3389/fpsyg.2023.1160046)
Supplement: Supplementary file 5 [file Table_5.docx]

**Table 5.** Urban vs. rural area

|  | | Rural_Urban | Country_area | STOP | KEEP | START |
| --- | --- | --- | --- | --- | --- | --- |
| Rural_Urban | Pearson Correlation | 1 | -.033 | -.087 | ***-.107**** | ***-.178***** |
|  | Sig. (2-tailed) |  | .605 | .173 | .095 | .005 |
|  | N |  | 246 | 246 | 246 | 246 |
| Country_area | Pearson Correlation |  | 1 | .064 | ***.238***** | .084 |
|  | Sig. (2-tailed) |  |  | .316 | .000 | .191 |
|  | N |  |  | 246 | 246 | 246 |

**. Correlation is significant at the 0.01 level (2-tailed).

*. Correlation is significant at the 0.1 level (2-tailed). In the rest of the cases, correlation is significant at the 0.1 level (2-tailed).
