# Supplementary material for: The expectations of generation Z regarding the university educational act in Romania: optimizing the didactic process by providing feedback
Source: Front Psychol. 2023 Sep 29;14:1160046. doi: 10.3389/fpsyg.2023.1160046 (PMC10572363; doi:10.3389/fpsyg.2023.1160046)
Supplement: Supplementary file 6 [file Table_6.docx]

**Table 6.** Results of the F-test

|  | STOP | | KEEP | | START | |
| --- | --- | --- | --- | --- | --- | --- |
|  | *Variable 1* | *Variable 2* | *Variable 1* | *Variable 2* | *Variable 1* | *Variable 2* |
| Mean | 0.020611 | 0.01233 | 0.070007 | 0.060799 | 0.010661 | -0.0085 |
| Variance | 0.002027 | 0.002441 | 0.002547 | 0.000989 | 0.002818 | 0.002828 |
| F | 0.830427 |  | 2.574803 |  | 0.996495 |  |
| P(F<=f) one-tail | 0.15209 |  | 0.00 |  | 0.49013 |  |
| F Critical one-tail | 0.742386 |  | 1.353365 |  | 0.742386 |  |
| Conclusion | Unequal variances | | Equal variances | | Unequal variances | |
