# Supplementary material for: The expectations of generation Z regarding the university educational act in Romania: optimizing the didactic process by providing feedback
Source: Front Psychol. 2023 Sep 29;14:1160046. doi: 10.3389/fpsyg.2023.1160046 (PMC10572363; doi:10.3389/fpsyg.2023.1160046)
Supplement: Supplementary file 7 [file Table_7.docx]

**Table 7.** Hypothesized Mean Difference

|  | STOP |  | KEEP |  | START |  |
| --- | --- | --- | --- | --- | --- | --- |
|  | Variable 1 | Variable 2 | Variable 1 | Variable 2 | Variable 1 | Variable 2 |
| Mean | 0.020611 | 0.01233 | 0.070007 | 0.060799 | 0.010661 | -0.0085 |
| Variance | 0.002027 | 0.002441 | 0.002547 | 0.000989 | 0.002818 | 0.002828 |
| Pooled Variance | - | - | 0.001838224 | | - | - |
| Hypothesized Mean Difference | 0 |  | 0 |  | 0 |  |
| t Stat | 1.362864 |  | 1.677451 |  | 2.817107 |  |
| P(T<=t) two-tail | 0.174276 |  | 0.094735 |  | 0.005256 |  |
| t Critical two-tail | 1.97047 |  | 1.969734 |  | 1.970067 |  |
