# Supplementary material for: The expectations of generation Z regarding the university educational act in Romania: optimizing the didactic process by providing feedback
Source: Front Psychol. 2023 Sep 29;14:1160046. doi: 10.3389/fpsyg.2023.1160046 (PMC10572363; doi:10.3389/fpsyg.2023.1160046)
Supplement: Supplementary file 8 [file Table_8.docx]

**Table 8.** Correlations between gender and pedagogical aspect.

|  | | Gender | Age |
| --- | --- | --- | --- |
| STOP | Pearson Correlation | .130*^*^ | .118* |
|  | Sig. (2-tailed) | .042 | .065 |
|  | N | 246 | 246 |
| KEEP | Pearson Correlation | -.141*^*^ | -.097 |
|  | Sig. (2-tailed) | .028 | .128 |
|  | N | 246 | 246 |
| START | Pearson Correlation | -.071 | .097 |
|  | Sig. (2-tailed) | .267 | .128 |
|  | N | 246 | 246 |

**. Correlation is significant at the 0.05 level (2-tailed).

**. Correlation is significant at the 0.1 level (2-tailed).In the rest of the cases, correlation is significant at the 0.1 level (2-tailed).*
