# Supplementary material for: The expectations of generation Z regarding the university educational act in Romania: optimizing the didactic process by providing feedback
Source: Front Psychol. 2023 Sep 29;14:1160046. doi: 10.3389/fpsyg.2023.1160046 (PMC10572363; doi:10.3389/fpsyg.2023.1160046)
Supplement: Supplementary file 9 [file Table_9.docx]

**Table 9.** F-test for variables.

|  | Variable 1 | Variable 2 |
| --- | --- | --- |
| Mean | 3.681818182 | 5 |
| Variance | 116.8939394 | 152.3809524 |
| Observations | 22 | 22 |
| df | 21 | 21 |
| F | 0.767116477 |  |
| P(F<=f) one-tail | 0.274450568 |  |
| F Critical one-tail | 0.479803022 |  |
