# Supplementary material for: The expectations of generation Z regarding the university educational act in Romania: optimizing the didactic process by providing feedback
Source: Front Psychol. 2023 Sep 29;14:1160046. doi: 10.3389/fpsyg.2023.1160046 (PMC10572363; doi:10.3389/fpsyg.2023.1160046)
Supplement: Supplementary file 10 [file Table_10.docx]

**Table 10.** The impact of remedial solutions.

|  | Variable 1 | Variable 2 |
| --- | --- | --- |
| Mean | 3.681818182 | 5 |
| Variance | 116.8939394 | 152.3809524 |
| Observations | 22 | 22 |
| Hypothesized Mean Difference | 0 |  |
| df | 41 |  |
| t Stat | -0.376780768 |  |
| P(T<=t) one-tail | 0.354139798 |  |
| t Critical one-tail | 1.682878002 |  |
| P(T<=t) two-tail | 0.708279597 |  |
| t Critical two-tail | 2.01954097 |  |
