# Supplementary material for: The expectations of generation Z regarding the university educational act in Romania: optimizing the didactic process by providing feedback
Source: Front Psychol. 2023 Sep 29;14:1160046. doi: 10.3389/fpsyg.2023.1160046 (PMC10572363; doi:10.3389/fpsyg.2023.1160046)
Supplement: Supplementary file 11 [file Table_11.docx]

**Table 11.** Correlations among variables and teachers and disciplines, SKS evaluation form

|  | Teacher | Discipline |
| --- | --- | --- |
| STOP_Educational_climate | 0.12 | 0.11 |
| Sig. | 0.07 | 0.09 |
| STOP_Evaluation | -.132^*^ | -0.11 |
| Sig. | 0.04 | 0.07 |
| STOP_Program | .198^**^ | .228^**^ |
| Sig. | 0.00 | 0.00 |
| STOP_Discipline | -.127^*^ | -0.12 |
| Sig. | 0.05 | 0.07 |
| STOP_It_is_perfect | 0.11 | 0.11 |
| Sig. | 0.09 | 0.07 |
| KEEP_Atitude | 0.12 | .146^*^ |
| Sig. | 0.07 | 0.02 |
| KEEP_Social_relations |  | 0.11 |
| Sig. |  | 0.08 |
| KEEP_Content | 0.12 | .140^*^ |
| Sig. | 0.05 | 0.03 |
| KEEP_Extracurricular_Activities | -.160^*^ | -.159^*^ |
| Sig. | 0.01 | 0.01 |
| START_Social_relations | .132^*^ | .131^*^ |
| Sig. | 0.04 | 0.04 |
| START_Educational_climate | 0.11 | 0.11 |
| Sig. | 0.09 | 0.10 |
| START_Extracurricular_Activities | -0.12 | -.143^*^ |
| Sig. | 0.05 | 0.03 |
| *. Correlation is significant at the 0.05 level (2-tailed). | | |
| **. Correlation is significant at the 0.01 level (2-tailed). | | |
| The rest have Sig. < 0.1 (2-tailed). | | |
