# Supplementary material for: The expectations of generation Z regarding the university educational act in Romania: optimizing the didactic process by providing feedback
Source: Front Psychol. 2023 Sep 29;14:1160046. doi: 10.3389/fpsyg.2023.1160046 (PMC10572363; doi:10.3389/fpsyg.2023.1160046)
Supplement: Supplementary file 12 [file Table_12.docx]

**Table 12.** Correlations among variables and teachers and disciplines, standard evaluation form

|  | | My presence at the didactic activity was | Teacher_has_encuraged_communication | Content_transmission |
| --- | --- | --- | --- | --- |
| Teacher | Pearson Correlation | .144^*^ |  | .106 |
|  | Sig. (2-tailed) | .024 |  | .096 |
|  | Pearson Correlation | .133^*^ | .105 | .116 |
| Discipline | Sig. (2-tailed) | .037 | .099 | .069 |
